# Post-Translational Modification β-Hydroxybutyrylation Regulates *Ustilaginoidea virens* Virulence

**DOI:** 10.1016/j.mcpro.2023.100616

**Published:** 2023-07-12

**Authors:** Xiaoyang Chen, Yuhang Duan, Zhiyong Ren, Taotao Niu, Qiutao Xu, Zhaoyun Wang, Lu Zheng, Yaohui Wang, Xiaolin Chen, Junbin Huang, Yuemin Pan

**Affiliations:** 1 Anhui Province Key Laboratory of Crop Integrated Pest Management, Anhui Agricultural University, Hefei, China; 2 The Key Lab of Plant Pathology of Hubei Province, Huazhong Agricultural University, Wuhan, China; 3 State Key Laboratory of Agricultural Microbiology, Huazhong Agricultural University, Wuhan, China; 4 National Key Laboratory of Crop Genetic Improvement, Huazhong Agricultural University, Wuhan, China; 5 Center for Excellence in Molecular Plant Sciences, Chinese Academy of Sciences, Shanghai, China

**Keywords:** histone deacetylase, lysine β-hydroxybutyrylation, post-translational modification, *Ustilaginoidea virens*, virulence

## Abstract

Lysine β-hydroxybutyrylation (K_bhb_) is an evolutionarily conserved and widespread post-translational modification that is associated with active gene transcription and cellular proliferation. However, its role in phytopathogenic fungi remains unknown. Here, we characterized K_bhb_ in the rice false smut fungus *Ustilaginoidea virens*. We identified 2204 K_bhb_ sites in 852 proteins, which are involved in diverse biological processes. The mitogen-activated protein kinase UvSlt2 is a K_bhb_ protein, and a strain harboring a point mutation at K72, the K_bhb_ site of this protein, had decreased UvSlt2 activity and reduced fungal virulence. Molecular dynamic simulations revealed that K72_bhb_ increases the hydrophobic solvent-accessible surface area of UvSlt2, thereby affecting its binding to its substrates. The mutation of K298_bhb_ in the septin UvCdc10 resulted in reduced virulence and altered the subcellular localization of this protein. Moreover, we confirmed that the NAD^+^-dependent histone deacetylases UvSirt2 and UvSirt5 are the major enzymes that remove K_bhb_ in *U. virens*. Collectively, our findings identify regulatory elements of the K_bhb_ pathway and reveal important roles for K_bhb_ in regulating protein localization and enzymatic activity. These findings provide insight into the regulation of virulence in phytopathogenic fungi *via* post-translational modifications.

## Highlights

• We identified 2204 K_bhb_ sites in 852 proteins in *Ustilaginoidea virens*.
• K72_bhb_ of UvSlt2 and K298_bhb_ of UvCdc10 regulated fungal virulence.
• The NAD^+^-dependent histone deacetylases UvSirt2 and UvSirt5 are the major enzymes that remove K_bhb_ in phytopathogenic fungi.

## Graphical Abstract

**Figure:**
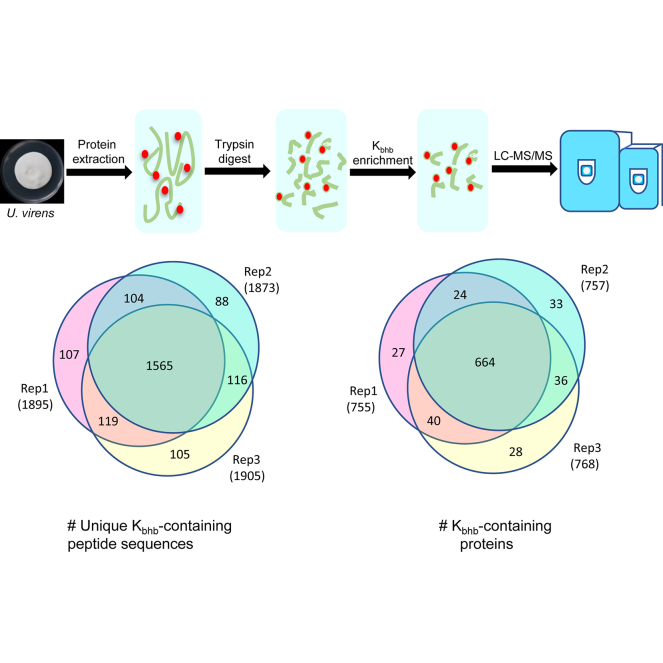

## In Brief

To explore the role of lysine β-hydroxybutyrylation (K_bhb_) in phytopathogenic fungi, we performed a proteomics analysis of this post-translational modification in the rice false smut fungus *Ustilaginoidea virens.* We identified 2204 β-hydroxybutyrylated sites in 852 proteins. K72_bhb_ affects the enzymatic activity of the mitogen-activated protein kinase UvSlt2, and K298_bhb_ affects the localization of the septin UvCdc10, thereby regulating the virulence of *U. virens*.

Chromatin structure and gene transcription are regulated by various post-translational modification (PTMs) of histones, such as methylation, acetylation, ubiquitination, and phosphorylation (1). PTMs also play important roles in protein localization, stability, and interactions, adding layers of complexity and flexibility to cellular signaling (2). Several PTMs, including acetylation, 2-hydroxyisobutyrylation, and crotonylation, function in the development and pathogenesis of phytopathogens (3, 4, 5). Over 300 types of PTMs have been identified in mammalian cells by mass spectrometry (MS) and pan-antibody enrichment (6, 7). However, few studies have focused on the roles of PTMs in the pathogenesis of phytopathogens.

Short-chain fatty acids are produced by cellular metabolism, serving as precursors for the generation of acyl-CoAs that can be used for acylations (8). Examples of these metabolites include α-ketoglutarate, butyrate, crotonate, and 3-hydroxybutyrate (9, 10, 11, 12, 13). Lysine β-hydroxybutyrylation (K_bhb_) is a recently identified acylation mark associated with active transcription (13). K_bhb_ is an evolutionarily conserved PTM among diverse eukaryotic species, including human embryonic kidney (HEK) 293 cells, mouse embryonic fibroblast cells, yeast (*Saccharomyces cerevisiae*) cells, and *Drosophila* S2 cells (13).

Forty-four K_bhb_ sites on histones were identified in HEK293 cells; increased H3K9_bhb_ is associated with the upregulation of genes that are involved in the starvation-response pathway (13). Based on MS data, K_bhb_ has been identified in both histone and nonhistone proteins, with transcription factors and a key metabolic enzyme being examples of the latter (14). MS analysis of the β-hydroxybutyrylome in HEK293 cells revealed 3248 K_bhb_ sites on 1397 proteins enriched for the functions chromatin remodeling, transcriptional regulation, and DNA repair (14). In mouse cells, 891 K_bhb_ sites were identified in 267 proteins by β-hydroxybutyrylome MS analysis, including proteins involved in amino acid, fatty acid, detoxification, and one-carbon metabolic pathways (15). K_bhb_ inhibits the activity of S-adenosyl-l-cysteine hydrolase, the rate-limiting enzyme in the methionine cycle, and altered metabolite levels, revealing that K_bhb_ affects hepatic metabolism under ketogenic conditions and demonstrating a functional consequence of this modification on a central metabolic enzyme (15). By contrast, the protein substrates bearing K_bhb_ in phytopathogens and their functions in various biological processes remain unknown.

Rice false smut, a disease caused by the pathogenic ascomycete fungus *Ustilaginoidea virens*, is a serious grain disease in rice-growing regions worldwide (16). This disease not only causes yield losses but also threatens human and animal health by producing cyclopeptide mycotoxins (16, 17, 18, 19). In this study, we explored the biological functions of K_bhb_ in *U. virens.* We identified 2204 K_bhb_ sites in 852 proteins using a proteomics approach. We also revealed that K_bhb_ plays key roles in multiple conserved cellular processes, protein localization, enzymatic activity, and the development and virulence of *U. virens*. This is the first study of the roles of K_bhb_ in fungal virulence.

## Experimental Procedures

### Fungal Strains, Culture, and Plant Inoculation

*U. virens* strain HWD-2 was used as the wildtype (20). HWD-2 and transformants derived from this strain were cultured on potato sucrose agar (PSA) at 28 °C in the dark. Liquid cultures prepared in potato sucrose broth (PSB) were incubated for 7 days with shaking at 180 rpm and homogenized in a blender to prepare mycelial and spore suspensions. Rice plants (*Oryza sativa* cv. Wanxian-98) were inoculated with 2 ml suspension *via* injection with a syringe in the middle section of each distal internode during the eighth stage of panicle development. Inoculate ten panicles for each strain in each experiment, and the plants were transferred to a greenhouse at 28 °C ± 2 deg. C with a relative humidity of 95 ± 5%. The experiment was repeated three times. About 30 inoculated panicles were collected at 21 days postinoculation for statistical analysis of the number of smut balls per panicle. *Asterisks* indicate significant differences using the unpaired Student’s *t* test (∗*p* < 0.05).

### Immunoblotting

Proteins were extracted from the samples and dissolved in 8 M urea. Protein concentration was measured with a BCA kit (Beyotime Biotechnology) according to the manufacturer’s instructions. The proteins were separated on SDS-PAGE gels and transferred to a polyvinylidene fluoride membrane using a Bio-Rad electroblotting apparatus, followed by immunoblotting with rabbit pan anti-K_bhb_ (PTM-1201RM) (1:1000 dilution; PTM Biolabs) primary antibody and anti-rabbit horseradish peroxidase secondary antibody (1:10,000 dilution; Sigma). The signals were detected using Pierce ECL Western blotting substrate (Thermo Fisher Scientific) in a ChemiDoc XRS+ system (Bio-Rad).

### Protein Extraction and Digestion

*U. virens* strain HWD-2 was cultured in PSB shaken at 180 rpm at 28 °C for 7 days, and the mycelia were collected to extract total protein. Frozen samples were ground to a fine powder in liquid nitrogen and combined with four volumes (compared with the volume of the sample) of lysis buffer (1% Triton X-100, 10 mM DTT, 3 μM tryptic soy agar, 50 mM n-acetylmuramic acid, and 1% protease inhibitor, 50 μM PR-619, 2 mM EDTA, and 1% phosphatase inhibitor for phosphorylation). The samples were lysed ultrasonically. An equal volume of Tris-saturated phenol (pH 8.0) was added, and the mixture was vortexed for 5 min. Following centrifugation (4 °C, 10 min, 5000*g*), the upper phenol phase was transferred to a new centrifuge tube. The proteins were precipitated by adding at least four volumes of ammonium sulfate–saturated methanol and incubated at −20 °C for at least 6 h. Following centrifugation at 4 °C for 10 min, the supernatant was discarded. The precipitate was washed with ice-cold methanol, followed by ice-cold acetone three times. The sample was dissolved in 8 M urea, and the protein concentration was determined with a BCA kit according to the manufacturer’s instructions.

For digestion, the volume of the protein solution was adjusted to that of lysis buffer. Trichloroacetic acid was slowly added to the sample to a final concentration of 20%. The sample was vortexed, mixed well, and precipitated at 4 °C for 2 h. Following centrifugation at 5000*g* for 5 min, the supernatant was discarded, and the precipitate was washed and precipitated 2 to 3 times with precooled acetone. The protein sample was diluted by adding 100 mM triethylamine bicarbonate to urea at a concentration of <2 M. Trypsin was added at a trypsin-to-protein mass ratio of 1:50 for the first overnight digestion. The reducing agent DTT was added to the sample to a final concentration of 5 mM, followed by incubation for 30 min at 56 °C. After adding iodoacetamide at a final concentration of 11 mM, the sample was incubated at room temperature (away from light) for 15 min. Finally, pooled samples were desalted through a Strata X C18 SPE column (Phenomenex) and dried by vacuum centrifugation.

### Fractionation and Enrichment of Lysine β-Hydroxybutyrylated Peptides

The tryptic peptides were dissolved in immunoprecipitation buffer (100 mM NaCl, 1 mM EDTA, 50 mM Tris–HCl, 0.5% NP-40, pH 8.0). To enrich for modified peptides, the tryptic peptides were dissolved in NETN buffer (100 mM NaCl, 1 mM EDTA, 50 mM Tris–HCl, 0.5% NP-40, pH 8.0) and incubated with prewashed K_bhb_ antibody beads (PTM-1204; PTM Bio) overnight at 4 °C with gentle shaking. The beads were washed for four times with NETN buffer and twice with water. The bound peptides were eluted from the beads with 0.1% trifluoroacetic acid. Finally, the eluted fractions were combined and vacuum dried. The resulting peptides were subjected to LC–tandem MS (MS/MS) after desalting using C18 ZipTips (Millipore) according to the manufacturer’s instructions.

### LC–MS/MS Analysis

The tryptic peptides were dissolved in solvent A (0.1% formic acid and 2% acetonitrile/in water) and loaded onto a home-made reverse-phase analytical column (25 cm length, 75/100 μm i.d.). The peptides were separated with a gradient from 6% to 22% solvent B (0.1% formic acid in acetonitrile) over 42 min, 22% to 30% in 12 min, and increasing to 30% to 80% in 3 min, then holding at 80% for the last 3 min, all at a constant flow rate of 450 nl/min on a nanoElute UHPLC system (Bruker Daltonics). The peptides were subjected to capillary HPLC followed by MS on a timsTOF Pro (Bruker Daltonics) system. The electrospray voltage was 2.0 kV. Precursors and fragments were analyzed in the TOF detector, with an MS/MS scan range from 100 to 1700 *m/z*. The timsTOF Pro was operated in parallel accumulation serial fragmentation mode. Precursors with charge states of 0 to 5 were selected for fragmentation, and ten parallel accumulation serial fragmentation–MS/MS scans were acquired per cycle. The dynamic exclusion was set to 30 s.

### Database Searches and Bioinformatic Analysis

The MS/MS data were searched with MaxQuant software (version 1.6.15.0) against the UniProt_*U. virens* protein database (www.ncbi.nlm.nih.gov/nuccore/JHTR00000000). Trypsin/P was specified as the cleavage enzyme, allowing a maximum of two missing cleavage sites. The mass tolerance for precursor ions was set to 20 ppm in the preliminary search and 5 ppm in the main search, and the mass tolerance for fragment ions was set to 0.02 Da. The false discovery rate (FDR) thresholds were <1% for peptides, proteins, and modification sites. Oxidized peptides were considered to be false positives and were removed from the list. Peptides identified from reverse or contaminating protein sequences were also removed from the list, as were peptides with a score <40, a site localization probability of <0.75, or when oxidized sites mapped to the C terminus of the peptide, unless the C terminus was the end of the corresponding protein.

Gene Ontology (GO) and protein–protein interaction analyses were performed using DAVID software (Database for Annotation, Visualization, and Integrated Discovery) and STRING (version 10.5). Sequence logos of significant motifs were generated with Motif-X software (version 5.0.2). Pathways were classified into hierarchical categories according to the Kyoto Encyclopedia of Genes and Genomes (KEGG) website. The subcellular localizations of the proteins were predicted using WoLF PSORT (version 0.2; https://wolfpsort.hgc.jp).

### Site-Directed Mutagenesis and Purification of UvSlt2 or UvCdc10

For site-directed mutagenesis, the native promoter region and full-length sequence of *UvSlt2* or *UvCdc10* were cloned into pNeo3300III-Flag. K119/K298 and K72 were assumed to be K_bhb_ sites of UvCdc10 and UvSlt2, respectively. Lysine was mutated to arginine (AAA-CGC), and sequences with single or double point mutations were obtained by PCR, followed by cloning into pNeo3300-Flag. *Agrobacterium tumefaciens* strain EHA105 cells with the complementary vectors were used to transform the conidia of the Δ*UvSlt2-1* or Δ*UvCdc10-1* mutant *via A. tumefaciens-*mediated transformation (21, 22). Transformants were selected on PSA supplemented with 500 μg/ml of the antibiotic G418. Transformants were confirmed by immunoblotting using anti-Flag antibody, including *UvSlt2-1* (Δ*UvSlt2-1* complementation strain), *UvSlt2*^*K72R*^*-1* (K72R point mutation strain), *UvCdc10-1* (Δ*UvCdc10-1* complementation strain), *UvCdc10*^*K119R*^*-1* (K119R point mutation strain), *UvCdc10*^*K298R*^*-1* (K298R point mutation strain), and *UvCdc10*^*K119R/K298R*^*-1* (K119R and K298R double point mutation strain). For immunoprecipitation, total proteins were extracted from the infiltrated strains and incubated with Anti-Flag M2 affinity gel (Yeasen Biotech). The conjugated beads were washed three times with PBS and incubated overnight with whole cell lysates at 4 ºC. Unbound proteins were removed from the beads *via* three washes with PBS. The beads containing bound proteins were boiled in SDS loading buffer for 5 min, and the proteins were subjected to immunoblotting.

### UvSlt2 Activity Assay

UvSlt2 activity assay was measured using an mitogen-activated protein kinase (MAPK) ELISA Kit (Abbexa), which is used to perform double-antibody one-step sandwich ELISA. The samples, standards, and horseradish peroxidase–labeled antibodies were placed in the microwells, which were precoated with MAPK antibody. The microwells were completely incubated and washed thoroughly with PBS buffer. Tetramethylbenzidine was used as a chromogenic substrate, and the intensity of the color was positively correlated with the mitogen-activated protein kinase activity in the sample. The absorbance value was measured with a microplate reader at 450 nm to calculate enzyme activity. All measurements were repeated ten times.

### Molecular Dynamics Simulations

The initial model of the UvSlt2 module was prepared from a publicly available crystal structure (Protein Data Bank code: 5Z33, 89.83% sequence identity) *via* structural modeling using MODELLER software (version 9.14). All simulations were based on the initial model, and seven systems were prepared for simulation using GROMACS (version 4.6) with the OPLS-AA/L all-atom force field. The protein was solvated in simple point charge water molecules in a cubic box; the edges of the box were located ∼1.0 nm from any atom of the protein. Additional Na^+^ and Cl^−^ ions were added to neutralize the charge of each system. Energy minimization of each system was performed using the steepest descent integrator setting until the maximum force reached <1000 kJ/mol/nm on any atom or until additional steps resulted in a potential energy change of <1 kJ/mol.

Simulations were then performed at a constant temperature of 300 K, and the V-rescale algorithm was used with a temperature coupling time constant of 0.1 ps. All bond lengths were constrained using the LINCS (linear constraint solver) algorithm. van der Waals interactions were measured using a simple cutoff of 1.4 nm, and long-range electrostatic interactions were handled using the particle mesh Ewald method with a fourth-order spline interpolation and a Fourier grid spacing of 0.1 nm. Once each system was sufficiently equilibrated around the target temperature, isotropic pressure coupling was performed, with the constant pressure set to 1.0 bar in all directions and a pressure coupling time constant of 1.0 ps. Finally, each system was subjected to 10 ns of molecular dynamics (MD) simulation; the time step used in the simulations was 2 fs. All analyses were performed using the GROMACS suite of tools and a secondary structure recognition algorithm (DSSP), as implemented in GROMACS. The final structures were visualized using the PyMOL Molecular Graphics System (version 1.7.2).

### Generation of GFP Fusion Constructs

A full-length UvCdc10 or MoSep4 gene–coding region and 2 kb native promoter were cloned into vector p3300neo-GFP. The EHA105 strain with the p3300neo-UvCdc10-GFP and p3300neo-MoSep4-GFP vector was transformed using *A. tumefaciens*-mediated transformation by coculture with conidia of the Δ*UvCdc10* and Δ*MoSep4* mutants, respectively. Subcellular localization of UvCdc10-GFP or MoSep4-GFP was photoed using a Zeiss LSM 510 Meta confocal fluorescence microscope, with the combinations of excitation and emission wavelengths of 488 nm/bandpass 500 to 550 nm for GFP.

### Protein Purification and *in Vitro* De-β-hydroxybutyrylation Assay

Full-length *UvRpd3*, *UvSirt2*, or *UvSirt5* complementary DNA was cloned into pET32a, which was used to transform BL21 (DE3)-competent *Escherichia coli* cells. Protein production was induced in the cells by adding 0.1 M IPTG. The cells were harvested, washed with PBS buffer, and disrupted by ultrasonication to extract the proteins. Recombinant UvRpd3-His, UvSirt2-His, or UvSirt5-His protein was purified with HisPur Ni–NTA Resin (Thermo Fisher Scientific). The histone protein or purified UvSlt2-Flag, UvCdc10-Flag protein of *U. virens*, and purified recombinant UvRpd3-His, UvSirt2-His, or UvSirt5-His protein were incubated in reaction buffer (50 mM Tris–HCl, 137 mM NaCl, 2.7 mM KCl, 1 mM MgCl_2_, and 1 mM DTT [pH 8.5]) at 30 °C for 4 h. The reaction products were analyzed by SDS-PAGE and immunoblotting using pan anti-K_bhb_ antibody.

### Experimental Design and Statistical Rationale

To determine the regulatory β-hydroxybutyrylated targets in *U. virens*, a qualitative β-hydroxybutyrylome analysis was performed. Three biological samples for *U. virens* mycelia were prepared and analyzed. The three samples were harvested, digested, and affinity enrichment were performed before LC–MS/MS analysis. The resulting MS/MS data were processed using the MaxQuant software (version 1.6.15.0). Tandem mass spectra were searched against *U. virens* database from UniProt. The FDR thresholds for protein, peptide, and modification sites were specified at 1%. The identified K_bhb_ proteins were enriched by GO enrichment and KEGG pathway analyses. A two-tailed Fisher’s exact test was employed to test the enrichment of the protein-containing international protein index entries against all international protein index proteins. Correction for multiple hypothesis testing was carried out using standard FDR control methods (set as 1%). The GO and KEGG pathways with corrected *p* values <0.001 were considered statistically significant. Lys-β-hydroxybutyrylated motifs were analyzed by motif-x at a significance level of 0.000001. The statistical tests used to analyze the data (growth rate, conidiation, biomass, enzyme activity, inoculation test, etc.) are indicated in the respective figures and/or article sections. The presence of different letters above the mean values of three replicates indicates a significant difference between different strains and samples (*p* < 0.05, ANOVA).

## Results

### β-hydroxybutyrate Affects the Mycelial Growth and Conidiation of *U. virens*

β-hydroxybutyrate is a precursor of K_bhb_. To determine whether β-hydroxybutyrate will directly inhibit the growth of *U. virens*, we compared the growth rate of *U. virens* on PSA medium containing different concentrations of β-hydroxybutyrate. The results showed that the mycelial growth rate of *U. virens* was significantly reduced with high concentration of β-hydroxybutyrate (supplemental Fig. S1, *A* and B). We then compared the conidiation and biomass of *U. virens* in PSB medium containing different concentrations of β-hydroxybutyrate; the conidiation of *U. virens* was significantly increased, whereas the biomass of *U. virens* significantly reduced with high concentration of β-hydroxybutyrate (supplemental Fig. S1, *C*–*E*). These results suggested that β-hydroxybutyrate treatment directly affects the mycelial growth and conidiation of *U. virens.*

### Identification of K_bhb_ Sites and Proteins in *U. virens*

**Fig. 1:**
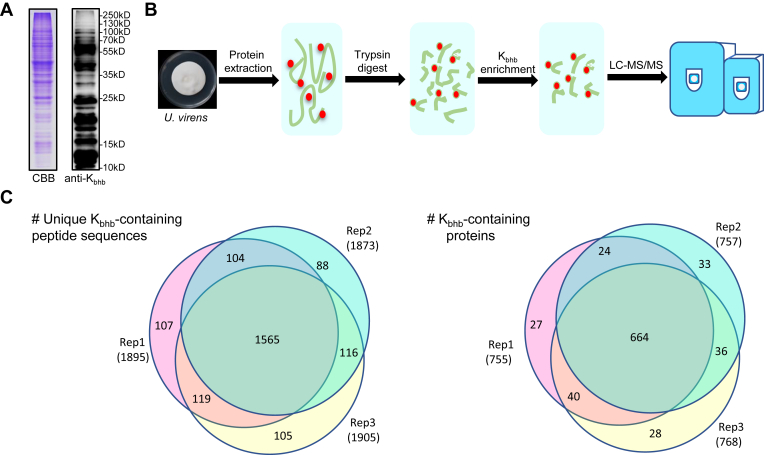
**Detection and site-resolved analysis of the K**_**bhb**_**status of proteins in *Ustilaginoidea virens*.***A*, Western blot analysis of K_bhb_ in *U. virens*. The protein was extracted using the TCA–acetone precipitation method. SDS-PAGE gel was stained with Colloidal Blue as the loading control. *B*, experimental workflow for the identification of K_bhb_ sites using a proteomics approach from 7-day cultured mycelia. *C*, overlap of K_bhb_ sites and proteins identified in three biological replicates. CBB, Coomassie Brilliant Blue; K_bhb_, lysine β-hydroxybutyrylation; TCA, trichloroacetic acid.

**Fig. 2:**
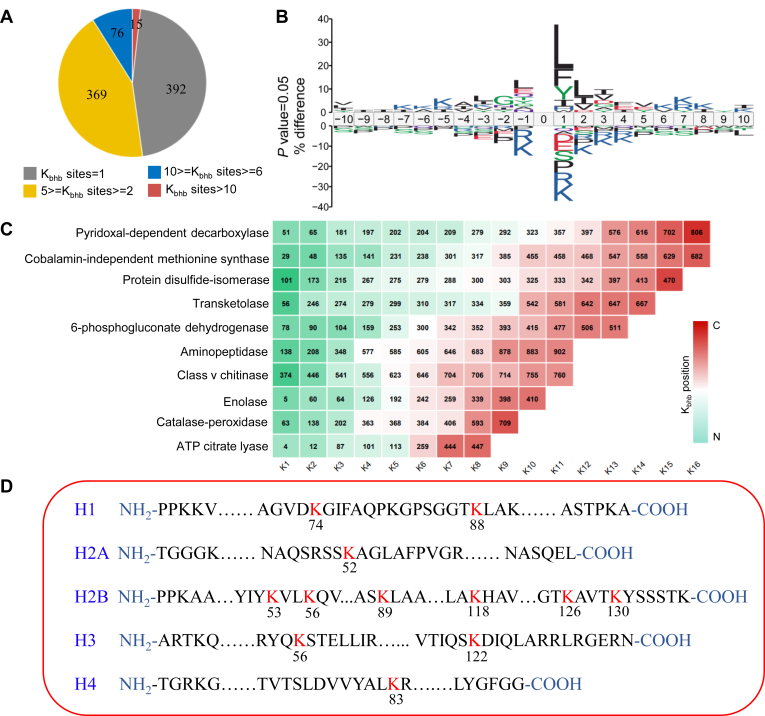
**Characterization of K**_**bhb**_**sites in *Ustilaginoidea virens*.***A*, distribution of K_bhb_ proteins based on number of modification sites. *B*, motif analysis of conserved amino acids flanking K_bhb_ sites. The size of an amino acid reflects the difference in the frequency of an amino acid in the experiment and its frequency in the reference set. The *p* value of each amino acid at every position was calculated by testing the experimental frequency against the frequency of each amino acid in the reference set with Fisher’s exact test. In this iceLogo, only significant amino acids (*p* < 0.05) are shown. *C*, graphical summary of metabolic enzymes with most K_bhb_ sites detected in this study. *Square number* represents number of K_bhb_ sites, whereas the number in each *square* shows position of lysine where the protein is β-hydroxybutyrylated. K_bhb_ positions close to N and C terminus of the proteins are marked as *blue* and *pink*, respectively. *D*, histone K_bhb_ sites were determined by mass spectrometry. K_bhb_, lysine β-hydroxybutyrylation.

To determine whether K_bhb_ is present in *U. virens*, we conducted immunoblotting to detect K_bhb_ in *U. virens* using specific pan-antibodies. We identified numerous protein bands with a wide range of molecular masses containing this modification (Fig. 1*A*). To identify protein substrates bearing K_bhb_, we carried out a proteomics screening using an affinity-directed MS method that involves tryptic digestion of extracted proteins isolated from mycelia following 7 days of culture in PSB medium, high-pH HPLC fractionation, affinity enrichment of K_bhb_ peptides with an anti-K_bhb_ antibody, and, finally, HPLC–MS/MS analysis and database searching (Fig. 1*B*). The mass errors for K_bhb_-containing peptides were <5 ppm, confirming that our MS data were highly accurate (supplemental Fig. S2*A*). Most identified peptides varied from 7 to 20 amino acids long (supplemental Fig. S2*B*). In total, 5665 β-hydroxybutyrylated peptides encompassing 2204 K_bhb_ sites from 852 proteins were identified at an FDR <1% (Fig. 1*C* and supplemental Table S1) with three independent repeated experiments. To examine the distribution of K_bhb_ sites in individual proteins, we calculated the number of these sites per protein. Whereas 392 proteins had only one modification site, 369 proteins had two to five modification sites, 76 proteins had 6 to 10 modification sites, and 15 proteins harbored ten modification sites (Fig. 2*A*).

To identify a possible consensus motif for K_bhb_, we compared the amino acid sequences surrounding these sites using iceLogo. With the positions of the β-hydroxybutyrylated lysine (K) residues defined as the 0 positions, we determined that lysine (K) residues were enriched from positions −7 to −5 and from positions +6 to +10. Similarly, leucine (L) was overrepresented at positions −3, −2, +1, and +2, whereas isoleucine (I) was enriched at the +1 to +3 positions (Fig. 2*B*). Lysines often occurred in vicinal dithiol structures with leucine; such LKLL motifs were located in various subcellular compartments (supplemental Fig. S3).

Notably, 371 of the 852 proteins harboring K_bhb_ marks were metabolic enzymes. Among these, 189 contained multiple K_bhb_ sites and eight were heavily modified (with >10 K_bhb_ sites). The most highly modified (K_bhb_) proteins were pyridoxal-dependent decarboxylase (Uv8b_6874), cobalamin-independent methionine synthase (Uv8b_5222), protein disulfide-isomerase (Uv8b_5623), transketolase (Uv8b_3056), 6-phosphogluconate dehydrogenase (Uv8b_5582), aminopeptidase (Uv8b_4979), class v chitinase (Uv8b_3637), enolase (Uv8b_2980), catalase-peroxidase (UV8b_3657), and ATP citrate lyase (Uv8b_5265) (Fig. 2*C*). We reasoned that K_bhb_ might affect the activities or functions of enzymes if this modification targets key residues.

Furthermore, we detected 12 K_bhb_ sites on histones, including H2A (K52), H2B (K53, K56, K89, K118, K126, and K130), H3 (K56 and K122), and H4 (K83) (Fig. 2*D*). Interestingly, histone H1 contained two K_bhb_ sites (K74 and K88); the presence of two target sites on a single protein has not been reported for other forms of PTM such as acetylation and 2-hydroxyisobutyrylation. K_bhb_ might function as a chromatin modification mark that epigenetically regulates target gene transcription in *U. virens*. Thus, we determined that K_bhb_ is an abundant and complex PTM in *U. virens*.

### Subcellular Localization, Functional Annotation, and Interaction Networks of K_bhb_ Proteins

**Fig. 3:**
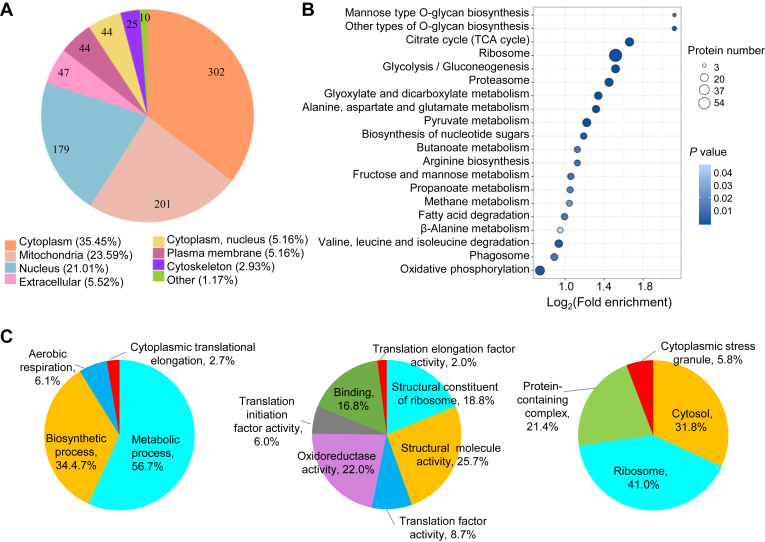
**Identification of subcellular localization and functional characterization of K**_**bhb**_**proteins in *Ustilaginoidea virens*.***A*, pie chart of subcellular localization of K_bhb_ proteins. *B*, KEGG enrichment analysis of K_bhb_ proteins identified in this study. *C*, Gene Ontology (GO) enrichment analysis of K_bhb_ proteins for the categories biological processes, molecular functions, and cellular components. K_bhb_, lysine β-hydroxybutyrylation; KEGG, Kyoto Encyclopedia of Genes and Genomes.

We also predicted the subcellular localizations of the K_bhb_ proteins. Most of the proteins were located in the cytoplasm (35.45%), mitochondria (23.59%), nucleus (21.01%), extracellular space (5.52%), or others (Fig. 3*A*). To identify which pathways may be affected by K_bhb_, we performed KEGG pathway enrichment analysis of the K_bhb_ proteins. Glycolysis/gluconeogenesis, ribosome, proteasome, and oxidative phosphorylation pathways as well as various metabolic pathways were significantly enriched (Fig. 3*B* and supplemental Table S2).

To further explore the functions of K_bhb_ proteins, we performed GO analysis. Enrichment analysis in the biological process category indicated that the K_bhb_ proteins were associated with various metabolic processes, biosynthetic processes, aerobic respiration, and cytoplasmic translational elongation (Fig. 3*C* and supplemental Table S3). Within the molecular function category, most K_bhb_ proteins were associated with structural molecule activity, oxidoreductase activity, structural constituent of ribosome, translation factor activity, translation initiation factor activity, and translation elongation factor activity (Fig. 3*C* and supplemental Table S3). The most highly enriched GO terms in the cellular components category were cytosol, ribosome, protein-containing complex, and cytoplasmic stress granule (Fig. 3*C* and supplemental Table S3).

To identify associations among K_bhb_ proteins in *U. virens*, we generated a protein interaction network of all these proteins using the STRING database. Some virulence- and stress response–related K_bhb_ proteins were present in the network, including ribosome structural constituents, translation initiation factors, proteasome, MAPK pathway proteins, septins, autophagy-related proteins, and endocytosis pathway proteins (supplemental Fig. S4, *A*–*G* and supplemental Table S4). These results reveal candidate proteins for further functional studies aimed at determining the potential roles of K_bhb_ in important biological processes in *U. virens*.

### Mutating the K_bhb_ Site in UvSlt2 Reduces Fungal Virulence and the Enzymatic Activity of this MAPK

**Fig. 4:**
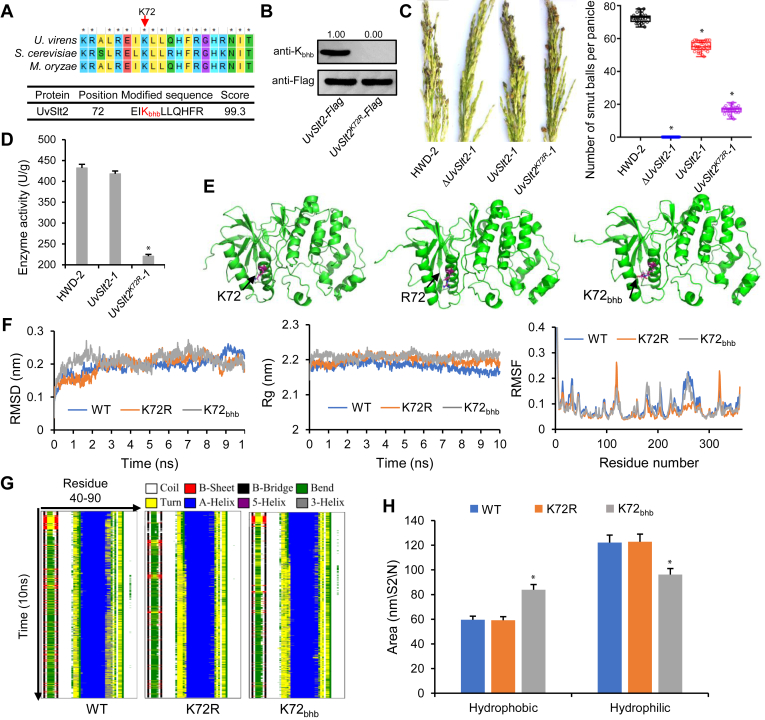
**UvSlt2 is a K**_**bhb**_**protein.***A*, MS/MS spectrum of *in vivo* K_bhb_ site of UvSlt2. *B*, the K_bhb_ levels of purified recombinant UvSlt2-Flag and UvSlt2^K72R^-Flag were determined by Western blotting using a pan anti-K_bhb_ and anti-Flag antibodies. *C*, *left*, virulence of the wildtype HWD-2, *ΔUvSlt2-1* mutant, complementation strain *UvSlt2-1*, and point mutant *UvSlt2*^*K72R*^*-1* at 21 dpi on rice cultivar, Wanxian-98. *Right*, average number of smut balls per panicle was calculated. *D*, K_bhb_ of K72 affected the enzymatic activity of UvSlt2. Data were collected from three independent experiments for each treatment and analyzed by Fisher’s least significant difference (LSD) test. *Asterisks* represent significant differences between mutant and WT by LSD at *p* = 0.05. *E*, final structures of the simulations of UvSlt2, Slt2^K72R^, and UvSlt2 β-hydroxybutyrylated at K72. *F*, analysis of the RMSDs, root mean square fluctuations (RMSFs), and *R*_g_s for the different UvSlt2 systems. *G*, secondary structure evolution of UvSlt2 and its mutants. The evolution in secondary structure at each frame was monitored using the dictionary of protein secondary structure (DSSP) algorithm. In the stripes, each pixel represents the secondary structure (color-coded) of a residue (40–90, x-dimension) at a given time in simulation (y-dimension). *H*, time evolution of the solvent-accessible surface area (SAS) calculated for the different systems that were simulated. K_bhb_, lysine β-hydroxybutyrylation.

One of the 852 β-hydroxybutyrylated proteins was the MAPK UvSlt2 (Fig. 4*A*). To further explore the roles of K_bhb_ of proteins in the virulence of plant pathogenic fungi, we performed site-directed mutagenesis of the K_bhb_ site in UvSlt2, which is located at K72 (Fig. 4*A* and supplemental Fig. S5). To confirm that K72 is indeed the K_bhb_ site, we generated an UvSlt2^K72R^ point mutant strain, in which the lysine in the K_bhb_ site was mutated to arginines (K-R). Sequences encoding normal and K-R UvSlt2 were fused with Flag, and the resulting constructs were expressed in the Δ*UvSlt2*-1 mutant. Immunoblotting of the Flag-enriched fraction indicated that K_bhb_ of UvSlt2 was completely abolished by this mutation (Fig. 4*B*), confirming that UvSlt2 is β-hydroxybutyrylated at this site.

To study the effect of K_bhb_ on the functions of UvSlt2, we compared virulence among wildtype strain HWD-2, the knockout strain Δ*UvSlt2-1*, the complementation strain (*UvSlt2-1*), and the single point mutant strain (*UvSlt2*^*K72R*^*-1*). The β-hydroxybutyrylation-deficient (K-R) strain *UvSlt2*^*K72R*^*-1* showed reduced virulence compared with the *UvSlt2-1* strain (Fig. 4*C*). To investigate the effect of K_bhb_ on the virulence of UvSlt2, we measured the enzymatic activity of UvSlt2 in these strains. The point mutation strain *UvSlt2*^*K72R*^*-1* showed significantly reduced UvSlt2 activity (Fig. 4*D*), implying that K_bhb_ modulates the enzymatic activity of UvSlt2.

To gain insight into the mechanisms by which K_bhb_ affects the enzymatic activity of UvSlt2, we performed MD simulations to examine how K_bhb_ of K72 influences the structure of UvSlt2 (supplemental Table S5). When the lysine residues at position K72 were mutated to R or β-hydroxybutyrylated, the protein structures were almost the same as that of unmodified UvSlt2 (Fig. 4*E* and supplemental Fig. S6). MD simulations of β-hydroxybutyrylated UvSlt2 (K72_bhb_) indicated that K_bhb_ of K72 resulted in structures that were nearly identical to that of unmodified UvSlt2 (Fig. 4*F*). Therefore, the secondary structures of the mutants and unmodified UvSlt2 were similar and had undergone minor changes from the starting states to the final states (Fig. 4*G*).

Because of the perturbed secondary structures of β-hydroxybutyrylated UvSlt2 (K72_bhb_) models and conformational fluctuations, these structures had a strong tendency to form hydrophobic solvent-accessible surfaces (Fig. 4*H*). The mutation of K to R did not alter the hydrophobic solvent-accessible surface area. Importantly, after UvSlt2 was β-hydroxybutyrylated, the hydrophobic and hydrophilic solvent-accessible surface area significantly increased but the hydrophilic solvent-accessible surface area significantly decreased. Therefore, K_bhb_ alters the functions of UvSlt2 *via* the self-regulation of its enzymatic activity, thereby affecting the binding of this enzyme to its substrates.

### K_bhb_ Regulates the Subcellular Localization and Virulence of the Septin UvCdc10

Septins are conserved proteins that form heteropolymeric complexes that regulate important cellular processes in phytopathogens, including cell division, septation, cytoskeleton organization, vesicle trafficking, and the maintenance of cell wall integrity (23, 24, 25). In *U. virens*, septins form a hetero-oligomeric complex composed of four core septins: Cdc3, Cdc10, Cdc11, and Cdc12 (21). Interestingly, all septins in *U. virens* were identified as potential K_bhb_ proteins, suggesting that K_bhb_ regulates the functions of septins.

**Fig. 5:**
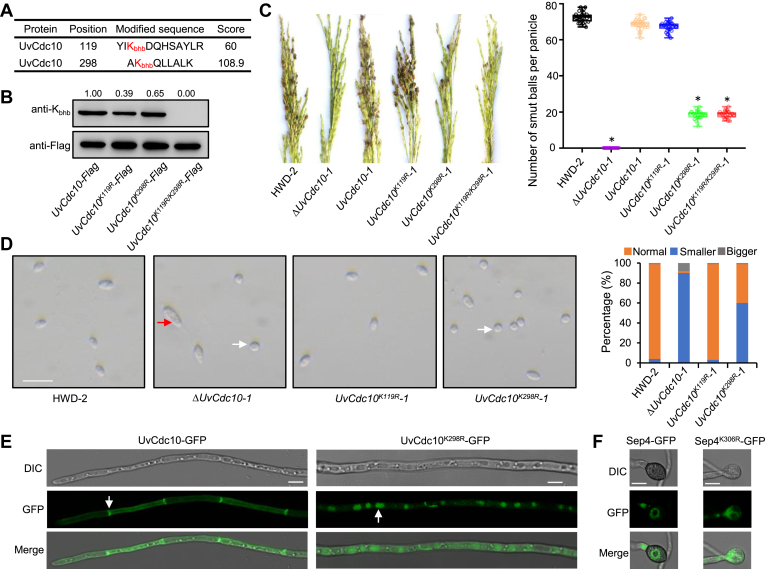
**Septin UvCdc10 is a K**_**bhb**_**protein.***A*, MS/MS spectrum of *in vivo* K_bhb_ site of UvCdc10. *B*, the K_bhb_ levels of purified recombinant UvCdc10-Flag, UvCdc10^K119R^-Flag, UvCdc10^K298R^-Flag, and UvCdc10^K119R/K298R^-Flag were determined by Western blotting using a pan anti-K_bhb_ and anti-Flag antibodies. *C*, *left*, virulence of the wildtype HWD-2, *ΔUvCdc10-1* mutant, complementation strain *UvCdc10-1* and point mutant *UvCdc10*^*K119R*^*-1*, *UvCdc10*^*K298R*^*-1*, and *UvCdc10*^*K119R/K298R*^*-1* strains at 21 dpi on rice cultivar, Wanxian-98. *Right*, average number of smut balls per panicle was calculated. *D*, *left*, conidia morphology of the wildtype HWD-2, *ΔUvCdc10-1* mutant, complementation strain *UvCdc10-1*, and point mutant *UvCdc10*^*K119R*^*-1*, *UvCdc10*^*K298R*^*-1*, and *UvCdc10*^*K119R/K298R*^*-1* strains in PSB for 7 days. *Red arrow*, bigger conidia; *white arrow*, smaller conidia; scale bar represents 20 μm. *Right*, increased numbers of globular conidia in the *UvCdc10*^*K298R*^*-1* mutants from 200 conidia. Bigger = abnormally enlarged conidia; Normal = oval conidia; Smaller = globular conidia. *E*, the subcellular localization of UvCdc10-GFP and UvCdc10^K298R^-GFP protein was observed in the vegetative hyphae of *Ustilaginoidea virens* under confocal microscopy. Scale bar represents 10 μm. *F*, the subcellular localization of Sep4-GFP and Sep4^K306R^-GFP protein was observed in the infection appressorium of *Magnaporthe oryzae* under confocal microscopy. Scale bar represents 10 μm. K_bhb_, lysine β-hydroxybutyrylation; PSB, potato sucrose broth.

To study the functional relevance of both septins and K_bhb_, we performed site-directed mutagenesis of the K_bhb_ sites in UvCdc10, which are located at K119 and K298 (Fig. 5*A* and supplemental Fig. S7). To confirm these sites, we generated the point mutant strains *UvCdc10*^*K119R*^*-1*, *UvCdc10*^*K298R*^*-1*, and *UvCdc10*^*K119R/K298R*^*-1*. Sequences encoding normal and K-R UvCdc10 constructs were fused with Flag, and the resulting constructs were expressed in the Δ*UvCdc10-1* mutant. Immunoblotting of the Flag-enriched fraction confirmed that K_bhb_ of UvCdc10 was seriously disrupted by these mutations (Fig. 5*B*), confirming that UvCdc10 is β-hydroxybutyrylated at these sites. To investigate the effect of K_bhb_ on the biological functions of UvCdc10, we compared virulence among the wildtype strain HWD-2, knockout strain Δ*UvCdc10-1*, complementation strain (*UvCdc10-1*), single point mutant (*UvCdc10*^*K119R*^*-1* and *UvCdc10*^*K298R*^*-1*), and double point mutant (*UvCdc10*^*K119R/K298R*^*-1*). The *UvCdc10*^*K298R*^*-1* and *UvCdc10*^*K119R/K298R*^*-1* strains showed reduced virulence compared with *UvCdc10-1* and *UvCdc10*^*K119R*^*-1* (Fig. 5*C*). These results indicate that K298_bhb_ plays key roles in the virulence of *U. virens*.

Septins are required to maintain normal cellular morphology in *U. virens* (21). To determine how K298_bhb_ affects the virulence of *UvCdc10*, we examined the conidial morphology of the HWD-2, Δ*UvCdc10-1*, *UvCdc10*^*K119R*^*-1*, and *UvCdc10*^*K298R*^*-1* strains by microscopy. The hyphae of the Δ*UvCdc10-1* and *UvCdc10*^*K298R*^*-1* strains were abnormally enlarged. Moreover, approximately 60% of the conidia of the *UvCdc10*^*K298R*^*-1* strain were globular in shape, whereas those of the HWD-2 and *UvCdc10*^*K119R*^*-1* strains were oval (Fig. 5*D*). These results indicate that K298_bhb_ plays important roles in the cellular morphology of *U. virens*.

Septins also play crucial roles in fungal infection structures. For example, in the rice blast fungus *Magnaporthe oryzae*, the septin ring helps organize the infection structure and allows it to breach the leaf surface (26). To investigate the role of K298_bhb_ in the subcellular localization of UvCdc10, we generated fusion constructs harboring GFP driven by the native promoter of *UvCdc10* or *UvCdc10*^*K298R*^ and separately transformed these constructs into the Δ*UvCdc10-1* mutant. In the UvCdc10-GFP transformant, strong GFP signals were observed in the septum and cytoplasm of vegetative hyphae by confocal microscopy. However, in the UvCdc10^K298R^-GFP transformant, UvCdc10^K298R^-GFP fusion protein was distributed in the cytoplasm, and it accumulated in the vacuole but not in the septum (Fig. 5*E*).

To further confirm that K_bhb_ regulates the subcellular localization of Cdc10, we introduced a K306R point mutation into Sep4 (UvCdc10 K298_bhb_ homologous site) in *M. oryzae* (supplemental Fig. S8). In the appressoria of *M. oryzae*, obvious septin rings were observed in the Sep4-GFP transformant, whereas in the Sep4^K306R^ transformant, no obvious septin rings were observed (Fig. 5*F*). These results indicate that K298_bhb_ plays core roles in regulating the subcellular localization of UvCdc10.

### UvSirt2 and UvSirt5 are Involved in Erasing Histone K_bhb_ and in the Virulence of *U. virens*

**Fig. 6:**
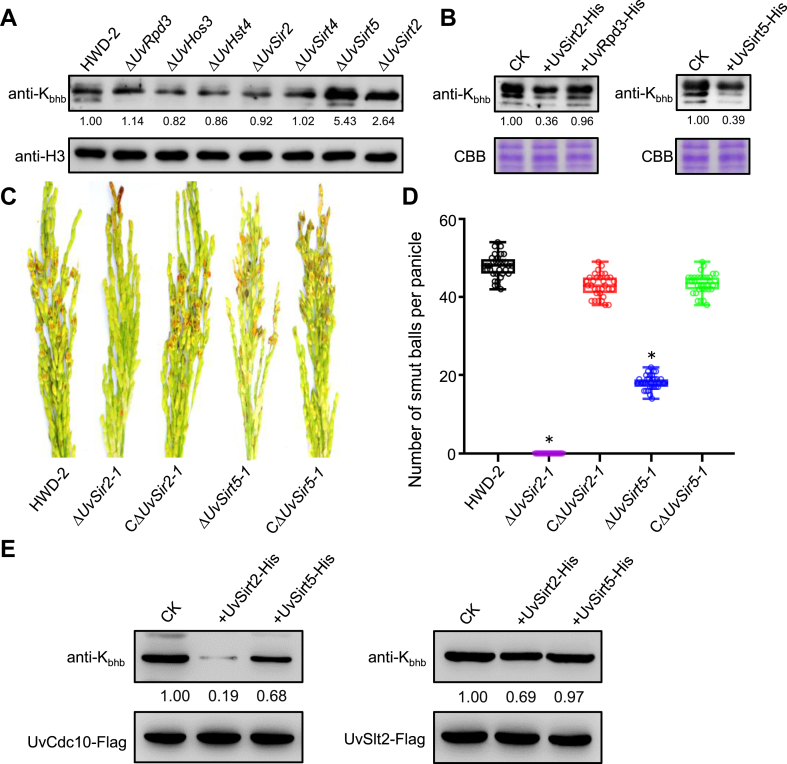
**UvSirt2 and UvSirt5 has a de-β-hydroxybutyrylase activity.***A*, histone K_bhb_ levels in six HDAC deletion mutants and the WT HWD-2 were detected by immunoblotting with a pan anti-K_bhb_ antibody. Total histones levels were visualized with anti-H3 antibody, and the relative signal intensity of each band is indicated, with the HWD-2 sample set to 1.00. *B*, *in vitro* lysine de-β-hydroxybutyrylation activity of UvRpd3, UvSirt2, and UvSirt5 by Western blotting assays with a pan anti-K_bhb_ antibody. Total histone levels were visualized with Coomassie blue staining. *C*, virulence of the WT HWD-2, *UvSirt2*, and *UvSirt5* deletion mutants and complementation strains at 21 dpi on rice cultivar, Wanxian-98. *D*, average number of smut balls per panicle. Data were collected from three independent experiments for each treatment and analyzed by Fisher’s least significant difference (LSD) test. *Asterisks* represent significant differences between mutant and WT by LSD at *p* = 0.05. *E*, Western blotting revealed that UvSirt2 and UvSirt2 can catalyze de-β-hydroxyisobutyrylation with UvSlt2 or UvCdc10. HDAC, histone deacetylase; K_bhb_, lysine β-hydroxybutyrylation.

Histone deacetylases (HDACs) are a family of enzymes that remove PTMs, such as acetylation, crotonylation, succinylation, 2-hydroxyisobutyrylation, butyrylation, formylation, and propionylation (27). To investigate whether HDACs regulate K_bhb_ in plant pathogenic fungi, we analyzed deletion mutants of six HDAC genes (including RPD3/HDA1 family genes [*UvRpd3* and *UvHos3*] and SIR2 family genes [*UvHst4*, *UvSirt2*, *UvSirt4*, and *UvSirt5*]) by immunoblot analysis using anti-K_bhb_ antibody. In the *UvSirt2* and *UvSirt5* deletion mutants, an increase in K_bhb_ was observed (Fig. 6*A*). To confirm this result, we expressed *UvSirt2*, *UvSirt5*, or *UvRpd3* in *E. coli* and obtained the purified proteins UvSirt2-His, UvSirt5-His, or UvRpd3-His. *In vitro* enzymatic activity assays showed that K_bhb_ levels were reduced in histone protein bands in response to UvSirt2-His or UvSirt5-His treatment, whereas K_bhb_ levels were unchanged in response to UvRpd3-His treatment (Fig. 6*B*), suggesting that UvSirt2 and UvSirt5 might serve as demodification enzymes to remove K_bhb_.

Finally, to investigate the roles of *UvSirt2* and *UvSirt5* in *U. virens* infection, we analyzed the virulence of *UvSirt2* and *UvSirt5* deletion mutants on susceptible rice cultivar Wanxian-98. At 21 days postinfection, compared with wildtype HWD-2 and the complementation strains, the Δ*UvSirt2* and Δ*UvSirt5* mutants produced significantly fewer false smut balls on spikelets (Fig. 6, *C* and *D*). To verify whether UvSirt2 or UvSirt5 can remove the K_bhb_ of UvSlt2 or UvCdc10, we performed *in vitro* experiments on purified UvSlt2 or UvCdc10. We found that UvSirt2 could remove the K_bhb_ of UvSlt2 and UvCdc10 and UvSirt5 could remove the K_bhb_ of UvSlt2 (Fig. 6*E*). These results demonstrate that UvSirt2 and UvSirt5 are important for K_bhb_ removal and for the virulence of *U. virens*.

## Discussion

Precursor proteins are typically inactive. A series of PTMs convert precursor proteins into mature functional proteins, thereby modulating their properties and functions (2). With the discovery of novel types of lysine PTMs, emerging data indicate that various modifications in eukaryotes are associated with both cellular physiology and pathogenesis (28, 29, 30, 31, 32, 33). The recently discovered histone mark K_bhb_ has been studied in mammalian cells (13, 14, 15), but to date, no studies of K_bhb_ in phytopathogens have been reported. In the current study, we performed the first proteomics analysis of K_bhb_ in the rice false smut fungus *U. virens.* β-hydroxybutyrylated proteins were enriched in the KEGG terms mannose type O-glycan biosynthesis, citrate cycle (tricarboxylic acid cycle), ribosome, glycolysis/gluconeogenesis, proteasome, glyoxylate and dicarboxylate metabolism, alanine, aspartate and glutamate metabolism, pyruvate metabolism, biosynthesis of nucleotide sugars, butanoate metabolism, arginine biosynthesis, fructose and mannose metabolism, propanoate metabolism, methane metabolism, fatty acid degradation, β-alanine metabolism, valine, leucine, and isoleucine degradation, phagosome, and oxidative phosphorylation pathway. These findings suggest that K_bhb_ is involved in the basic life activities of *U. virens*.

We identified several well-known infection-related proteins in *U. virens* as putative β-hydroxybutyrylated proteins. Several known virulence factors were modified by K_bhb_, including the MAPK pathway kinases UvSlt2 and UvCDC2, the septin UvCdc3-12, and the autophagy-related protein UvATG8 (33, 34, 35). To confirm the role of K_bhb_ in fungal virulence, we targeted the MAPK pathway kinase UvSlt2 for mutagenesis. Interestingly, disrupting K_bhb_ in UvSlt2 led to reduced virulence. Indeed, the Slt2-MAPK pathway plays conserved roles in pathogenesis in filamentous fungal pathogens (36). Point mutation of the K_bhb_ site of UvSlt2 significantly decreased its enzymatic activity and altered phosphorylation by the Slt2-MAPK pathway to regulate the virulence of *U. virens*.

Septins play important roles in the infection structures of the phytopathogen *M. oryzae.* The septin ring structure provides a scaffold for the F-actin ring, which organizes the exocyst and recruits it to the appressorial pore. The penetration peg emerges through the penetration pore to breach the surface of the rice leaf (26). However, the infection strategy of *U. virens* is quite different from that of other fungi. *U. virens* colonizes rice spikelets without forming specialized infection structures (such as appressoria or hyphal necks). Instead, only the hyphae of *U. virens* penetrate rice spikelets. *U. virens* septin deletion mutants failed to cause typical false smut symptoms (21). In the current study, we showed that the *U. virens*/*M. oryzae* septin UvCdc10/Sep4 can be modified by K_bhb_. We also identified the K_bhb_ site of the septin UvCdc10. More importantly, we demonstrated that K_bhb_ of K298 regulates the activities of septins by affecting their localization to the hyphae of *U. virens* or to the appressorial septin ring of *M. oryzae*, which is important for infection. This regulatory mechanism has not previously been demonstrated in plant pathogenic fungi, thus representing a new mechanism for infection.

A recent genomic analysis suggested that *U. virens* encodes at least 628 potential secreted proteins, 193 of which are putative effectors (37). An increasing number of effectors have been shown to play critical roles in the infection process of *U. virens*. We determined that 42 putative secreted proteins are β-hydroxybutyrylated proteins, including Uv8b_1036, Uv8b_1044, Uv8b_1061, Uv8b_1189, Uv8b_1932, Uv8b_1941, Uv8b_2375, Uv8b_2468, Uv8b_2607, Uv8b_2660, Uv8b_2727, Uv8b_273, Uv8b_3029, Uv8b_3274, Uv8b_3284, Uv8b_3682, Uv8b_3709, Uv8b_3724, Uv8b_3946, Uv8b_4172, Uv8b_4324, Uv8b_4753, Uv8b_5228, Uv8b_5521, Uv8b_5623, Uv8b_5731, Uv8b_5833, Uv8b_5917, Uv8b_6036, Uv8b_6361, Uv8b_6458, Uv8b_6660, Uv8b_6932, Uv8b_7147, Uv8b_7418, Uv8b_7648, Uv8b_7731, Uv8b_7754, Uv8b_7779, Uv8b_8142, Uv8b_8155, and Uv8b_8307. Forty genes encoding these secreted proteins were differentially expressed at four distinct infection stages in *U. virens* (supplemental Fig. S9), suggesting that these secreted proteins might be important for *U. virens* infection.

Interestingly, we determined that the subtilase UvPR1H (Uv8b_8155) is a β-hydroxybutyrylated effector. Subtilases play wide range of physiological roles, from nonspecific protein degradation to the formation of specific polypeptide hormones, as well as the degradation of proproteins to form active mature proteins (38). Eight subtilisin genes were identified in the genome of *U. virens*. Among these, *UvPr1a* and *UvPR1H* affect mycelial growth and the conidiation and pathogenicity of *U. virens.* UvPr1a directly targets and degrades the rice protein OsSGT1 (SUPPRESSOR OF G2 ALLELE OF *skp1*), thereby interfering with host immunity and promoting infection (39). UvPr1H is a β-hydroxybutyrylated virulence effector, but whether the K_bhb_ of UvPr1H affects its degradation activity to regulate the virulence of *U. virens* remains unknown.

In addition, the effector UvCBP1 (Uv8b_3637) is a β-hydroxybutyrylated protein. UvCBP1 (Chitin-Binding Protein) competes with the rice chitin receptor OsCEBiP to bind to chitin, thereby suppressing pattern-triggered immunity (40). UvCBP1 also interacts with the rice scaffold protein OsRACK1A, competing for its interaction with the NADPH oxidase OsRBOHB. This leads to a decrease in the phosphorylation level of OsRBOHB, thereby reducing the production of reactive oxygen species to increase the susceptibility of rice to pathogens (41). The role of K_bhb_ of core effectors in mediating the arms race of “pathogen attack and plant defense” in the rice–*U. virens* interaction system requires further investigation.

Histone lysine acylation is not only influenced by metabolic substrates but also is regulated by “readers,” “writers,” and “erasers.” Histone acetyltransferases (HATs) and HDACs catalyze acylation reactions for different PTMs (42, 43, 44). The acyltransferase p300 acts as a histone K_bhb_ writer by catalyzing the addition of 3-hydroxybutyrate to lysine. The levels of histone K_bhb_ at the H3K9, H3K18, H3K27, and H4K8 sites decreased in response to the knockdown of p300; some of these histone K_bhb_ sites were more sensitive to p300 knockdown than the corresponding K_ac_ sites (14, 45). Unfortunately, we did not obtain a knockout mutant of p300 in *U. virens*. Therefore, we used the pan-K_bhb_ antibody to analyze the levels of histone K_bhb_ in other histone acetylase mutants (including mutants of the MYST family HAT and GNAT family HAT), finding that these histone acetylases were not K_bhb_ writers (supplemental Fig. S10). Therefore, the writers of K_bhb_ in phytopathogens require further exploration.

In the past several years, classically annotated HDACs were shown to function in acetylation and other acetylation-independent acylations (46, 47, 48). In mammalian cells, the major “erasers” of histone K_bhb_ include zinc-dependent HDAC1 and HDAC2 and NAD-dependent SIRT3, which mediate the removal of K_bhb_ *via* their enzymatic activity (14, 45). In the current study, the NAD^+^-dependent protein deacetylases UvSirt2 and UvSirt5 were confirmed to be the major enzymes that remove K_bhb_ in *U. virens*. “Reader” proteins specifically recognize certain PTMs, which in turn regulate downstream gene transcription and influence various cellular events. Proteins containing bromodomains, YEATS (Yaf9, ENL, AF9, Taf14, and Sas5) domains, and double plant homeodomain finger domains were found to be “readers” that recognize lysine residues of histones (49). However, no unique reader proteins for K_bhb_ were previously identified. Whether double plant homeodomain finger– or YEATS-mediated K_bhb_ recognition is conserved across species and whether any other domain may be involved in K_bhb_ recognition require further investigation.

Recently, several types of new lysine acylation modifications were identified on histones. These lysine acylation reactions appear to use their corresponding short-chain CoAs as cofactors in the acylation reactions, suggesting additional mechanisms that link cellular metabolism and epigenetic regulation (13). Diverse lysine acylations such as histone acetylation (K_ac_), butyrylation (K_bu_), 2-hydroxyisobutyrylation (K_hib_), crotonylation (K_cr_), isobutyrylation (K_ibu_), and β-hydroxybutyrylation are associated with chromatin organization, metabolism, and so on in various cellular systems (9, 10, 11, 12, 13, 50). A fundamental question in this field is how the lysine acylation pathways are differentially regulated. In order to finely regulate these processes, there are complex crosstalk between different PTMs and histone-modifying enzymes. For example, K_ac_ has been considered to be one of the most extensive PTMs and plays key roles in various biological processes. Based on the previous studies, many proteins could undergo K_bhb_ and K_ac_ on the same site (3, 4). However, the functions of K_ac_ and K_bhb_ on the same K residue are unclear, and whether histone K_bhb_ has similar functions to K_ac_ in gene expression needs further research.

In summary, our findings increase our understanding of the roles of K_bhb_ in regulating the virulence of filamentous fungi and pave the way for further exploring the diverse biological functions of this modification.

## Data Availability

The MS proteomics data have been deposited to the ProteomeXchange Dataset (PRIDE) under the accession number PXD039304.

## Supplemental Data

This article contains supplemental data.

## Conflict of interest

The authors declare no competing interests.

## Supplemental Data

Supplemental Figure S1 **β-hydroxybutyrate affects the mycelial growth and conidiation of *U. virens.****A*, colonies of the wild-type strain HWD-2 on PSA medium containing different concentrations of β-hydroxybutyrate after 14 d at 28 °C. *B*, colonies diameter of the wild-type strain HWD-2 on PSA medium containing different concentrations of β-hydroxybutyrate after 14 d at 28 °C. *C*–*E*, vegetative growth and conidial production of the wild-type strain HWD-2 grown in PSB medium containing different concentrations of β-hydroxybutyrate at 180 rpm for 7 days.

Supplemental Figure S2 *A*, mass error of identified K_bhb_ peptides. *B*, distribution of peptide length detected with K_bhb_ sites by MS.

Supplemental Figure S3 Motif analysis for K_bhb_ sites in the cytoplasm, the nucleus, extracellular and mitochondria with respective proteins identified in sub-organelles as background. The numbers −10 and +10 indicate the upstream and downstream flanking sequences of cysteines (number 0), respectively.

Supplemental Figure S4 **Interaction networks of K**_**bhb**_**proteins in *U. virens*.***A*, 65 ribosome-associated proteins. *B*, 17 proteins associated with the translation initiation factors. *C*, 22 proteins associated with the proteasome. *D*, 10 proteins associated with the MAPK pathway. *E*, four proteins associated with septins. *F*, 15 proteins associated with the autophagy. *G*, 16 proteins associated with the endocytosis.

Supplemental Figure S5 **MS/MS spectra of a tryptic peptide for the UvSlt2_K72 β-hydroxybutyrylated peptide EIK**_**bhb**_**LLQHFR**.

Supplemental Figure S6 Final structures of stimulations of UvSlt2 at K72R (*A*) and K72_bhb_ (*B*).

Supplemental Figure S7 MS/MS spectra of a tryptic peptide for the UvCdc10_K119 β-hydroxybutyrylated peptide YIK_bhb_DQHSAYLR and UvCdc10_K298 β-hydroxybutyrylated peptide AK_bhb_QLLALK.

Supplemental Figure S8 **Homology analysis of Cdc10p in *M. oryzae* and *U. virens*.** Amino acid sequence alignment of UvCdc10 and MoSep4. Red highlights indicate the conserved regions of these proteins.

Supplemental Figure S9 RNA-seq analysis of the expression levels of genes coding β-hydroxybutyrylated effectors during the course of *U. virens* infection. S1, 1 dpi; S2, 3 dpi; S3, 6 dpi; S4, 15 dpi.

Supplemental Figure S10 Histone K_bhb_ levels in 7 HDAs deletion mutants and the wild-type HWD-2 were detected by immunoblotting with a pan anti-K_bhb_ antibody. Total histones levels were visualized with anti-H3 antibody, and the relative signal intensity of each band is indicated, with the HWD-2 sample set to 1.00.

Supplemental Table S1 Modification site information of K_bhb_ proteins in *U. virens*.

Supplemental Table S2 Gene Ontology enrichment of K_bhb_ proteins in *U. virens*.

Supplemental Table S3 KEGG pathway enrichment of K_bhb_ proteins in *U. virens*.

Supplemental Table S4 Detailed list of protein-protein interaction (PPI) map of K_bhb_ proteins identified in *U. virens*.

Supplemental Table S5 Data of molecular dynamics (MD) simulations of K72_bhb_ influences the structure of UvSlt2.
