# Supplementary figures and images for: Post-Translational Modification β-Hydroxybutyrylation Regulates Ustilaginoidea virens Virulence
Source: Mol Cell Proteomics. 2023 Jul 12;22(8):100616. doi: 10.1016/j.mcpro.2023.100616 (PMC10423879; doi:10.1016/j.mcpro.2023.100616)

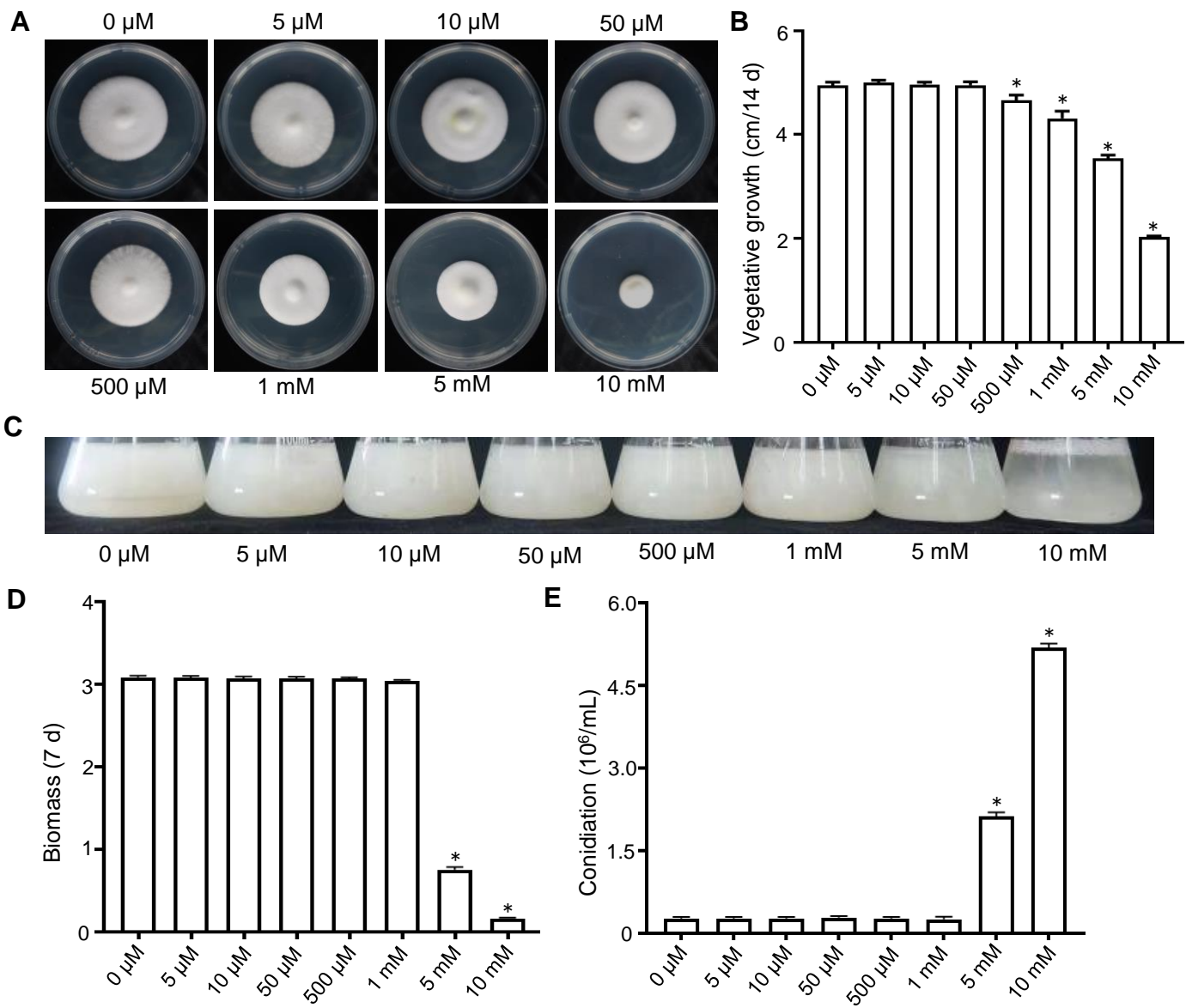

Supplement: Supplemental Figure S1 — β-hydroxybutyrate affects the mycelial growth and conidiation of U. virens.A, colonies of the wild-type strain HWD-2 on PSA medium containing different concentrations of β-hydroxybutyrate after 14 d at 28 °C. B, colonies diameter of the wild-type strain HWD-2 on PSA medium containing different concentrations of β-hydroxybutyrate after 14 d at 28 °C. C–E, vegetative growth and conidial production of the wild-type strain HWD-2 grown in PSB medium containing different concentrations of β-hydroxybutyrate at 180 rpm for 7 days. [file mmc1.pdf]

**A**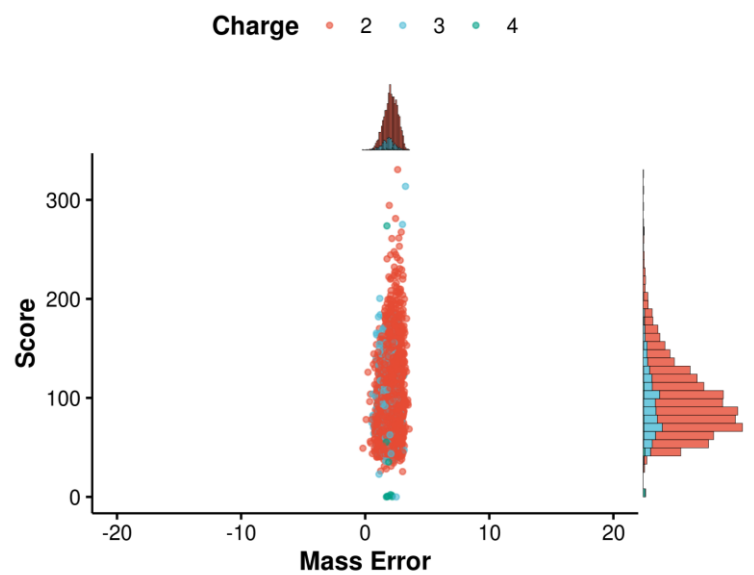**B**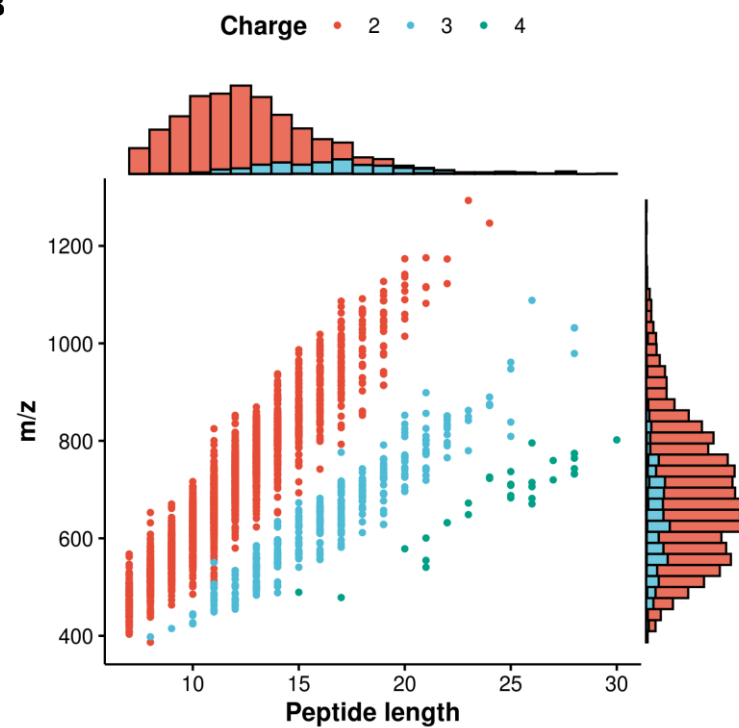

Supplement: Supplemental Figure S2 — A, mass error of identified Kbhb peptides. B, distribution of peptide length detected with Kbhb sites by MS. [file mmc2.pdf]

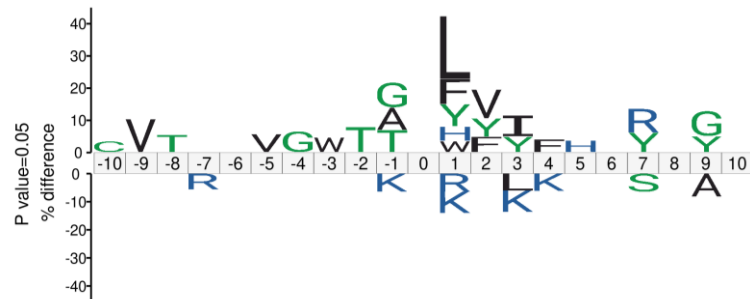

Extracellular

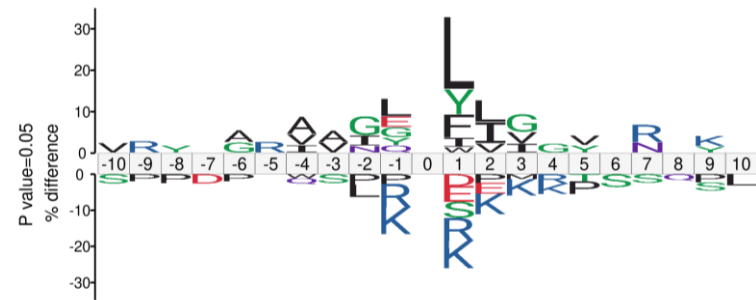

Mitochondria

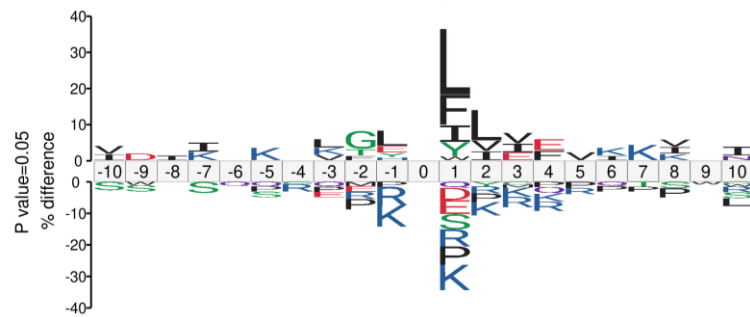

Cytoplasm

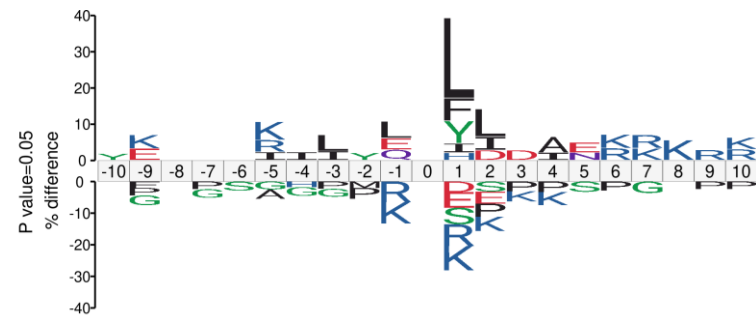

Nucleus

Supplement: Supplemental Figure S3 — Motif analysis for Kbhb sites in the cytoplasm, the nucleus, extracellular and mitochondria with respective proteins identified in sub-organelles as background. The numbers −10 and +10 indicate the upstream and downstream flanking sequences of cysteines (number 0), respectively. [file mmc3.pdf]

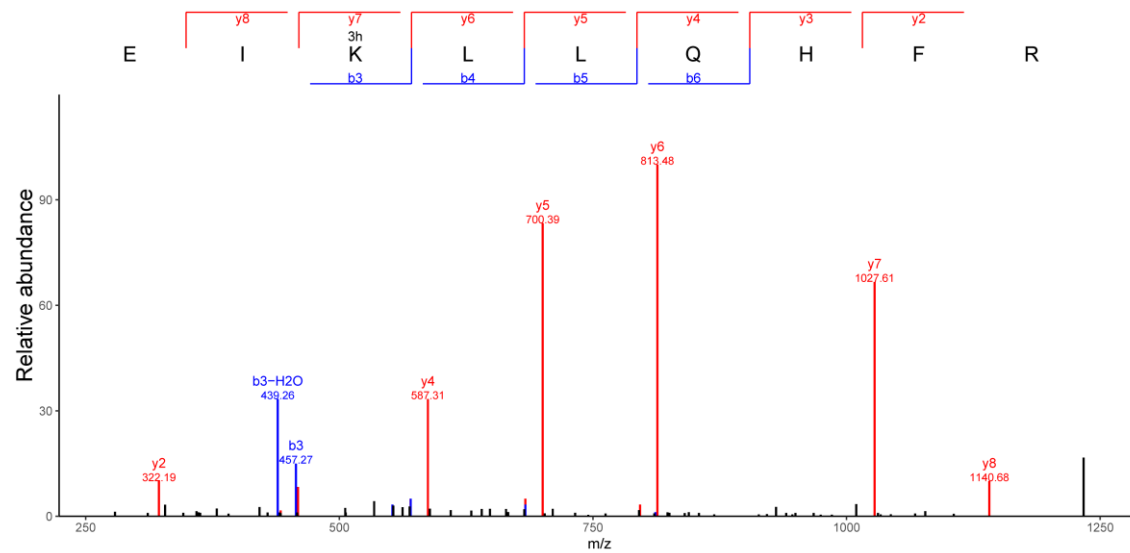

Supplement: Supplemental Figure S5 — MS/MS spectra of a tryptic peptide for the UvSlt2_K72 β-hydroxybutyrylated peptide EIKbhbLLQHFR. [file mmc5.pdf]

**A**

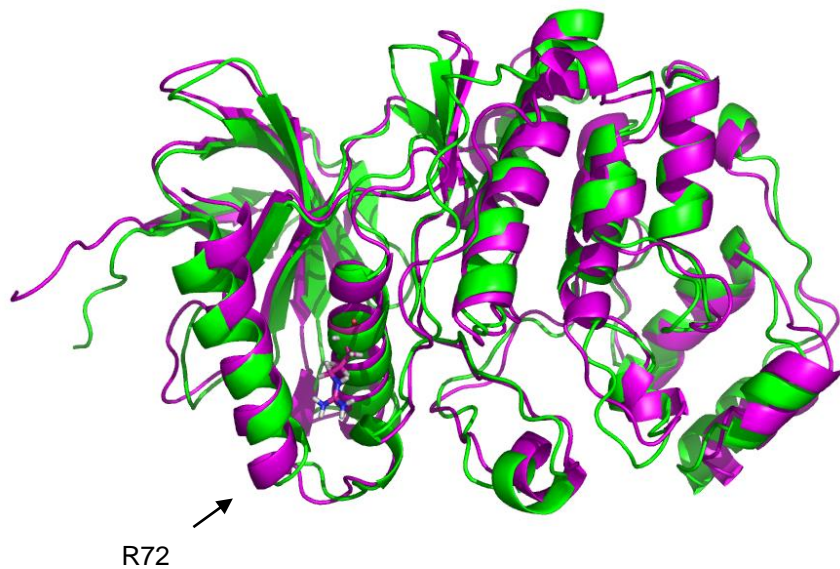

**B**

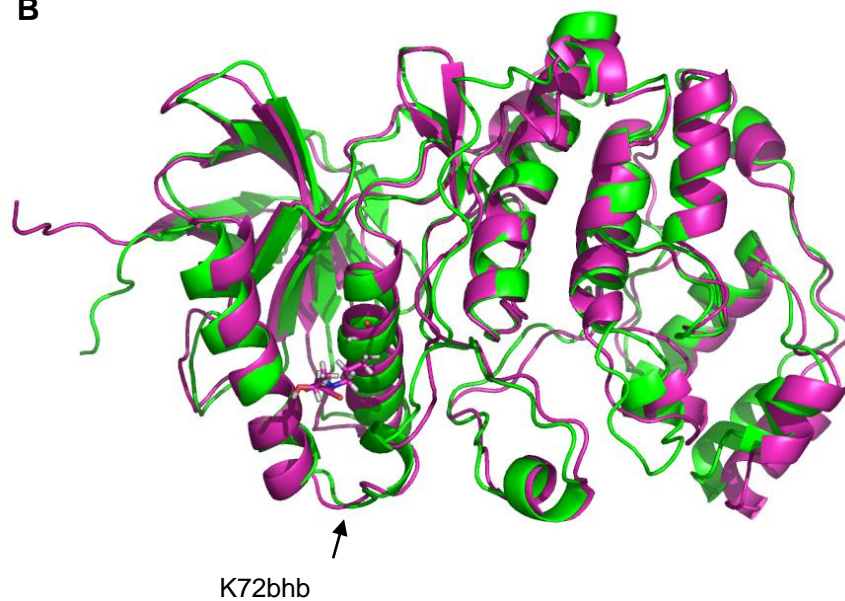

Supplement: Supplemental Figure S6 — Final structures of stimulations of UvSlt2 at K72R (A) and K72bhb (B). [file mmc6.pdf]

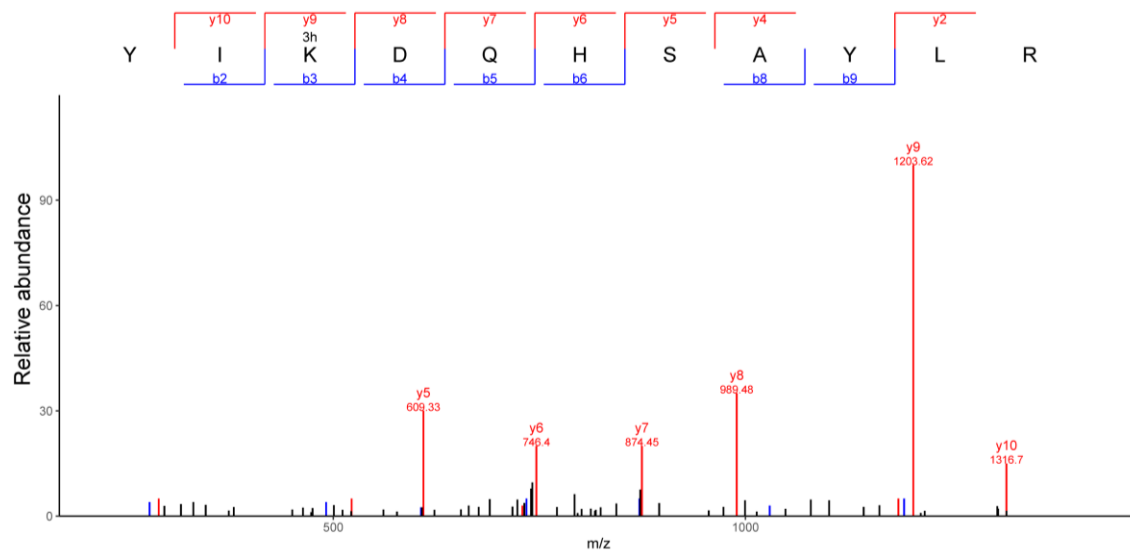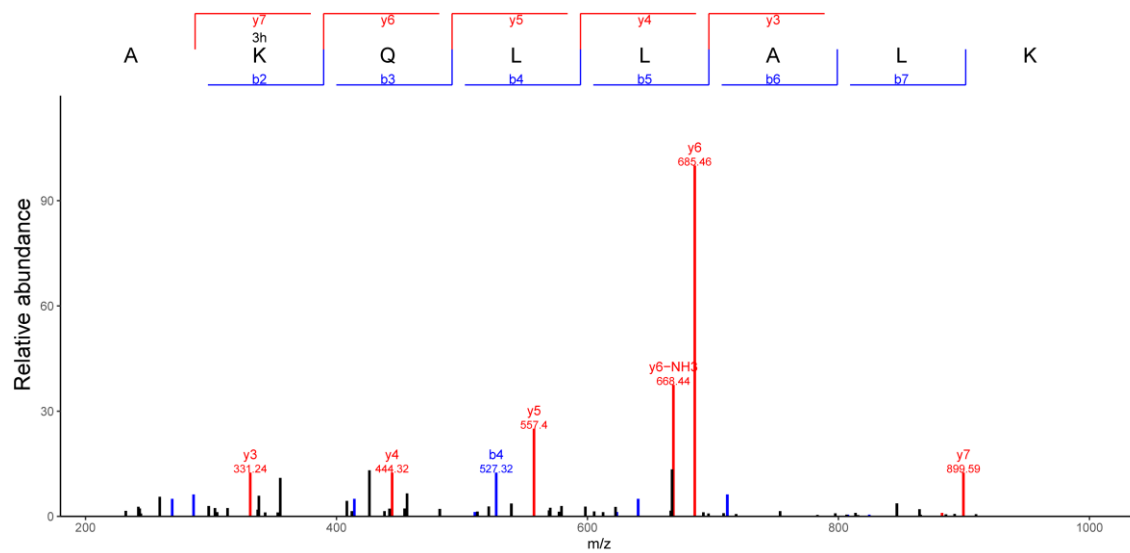

Supplement: Supplemental Figure S7 — MS/MS spectra of a tryptic peptide for the UvCdc10_K119 β-hydroxybutyrylated peptide YIKbhbDQHSAYLR and UvCdc10_K298 β-hydroxybutyrylated peptide AKbhbQLLALK. [file mmc7.pdf]

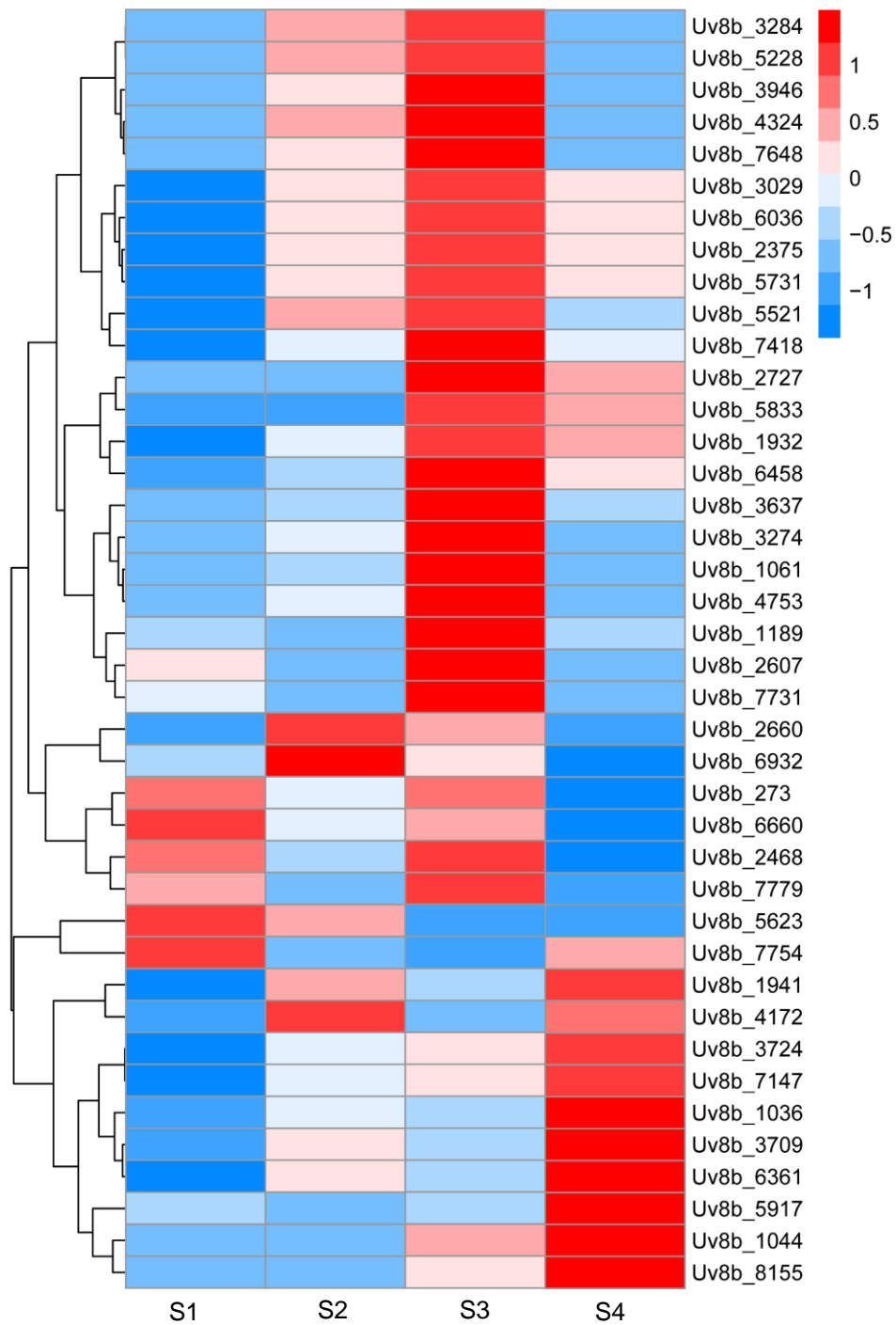

Supplement: Supplemental Figure S9 — RNA-seq analysis of the expression levels of genes coding β-hydroxybutyrylated effectors during the course of U. virens infection. S1, 1 dpi; S2, 3 dpi; S3, 6 dpi; S4, 15 dpi. [file mmc9.pdf]

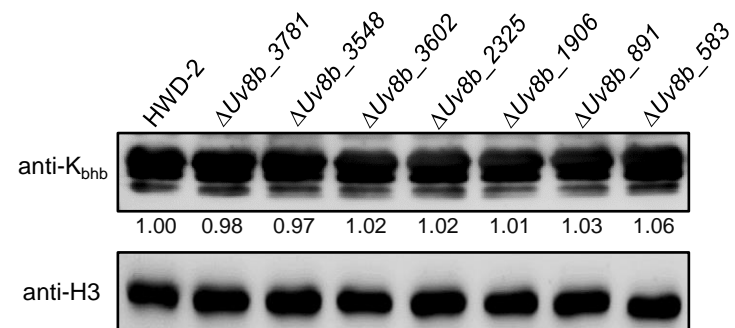

Supplement: Supplemental Figure S10 — Histone Kbhb levels in 7 HDAs deletion mutants and the wild-type HWD-2 were detected by immunoblotting with a pan anti-Kbhb antibody. Total histones levels were visualized with anti-H3 antibody, and the relative signal intensity of each band is indicated, with the HWD-2 sample set to 1.00. [file mmc10.pdf]
