# Supplementary material for: Post-Translational Modification β-Hydroxybutyrylation Regulates Ustilaginoidea virens Virulence
Source: Mol Cell Proteomics. 2023 Jul 12;22(8):100616. doi: 10.1016/j.mcpro.2023.100616 (PMC10423879; doi:10.1016/j.mcpro.2023.100616)
Supplement: Supplemental Figure S4 — Interaction networks of Kbhbproteins in U. virens.A, 65 ribosome-associated proteins. B, 17 proteins associated with the translation initiation factors. C, 22 proteins associated with the proteasome. D, 10 proteins associated with the MAPK pathway. E, four proteins associated with septins. F, 15 proteins associated with the autophagy. G, 16 proteins associated with the endocytosis. [file mmc4.pdf]

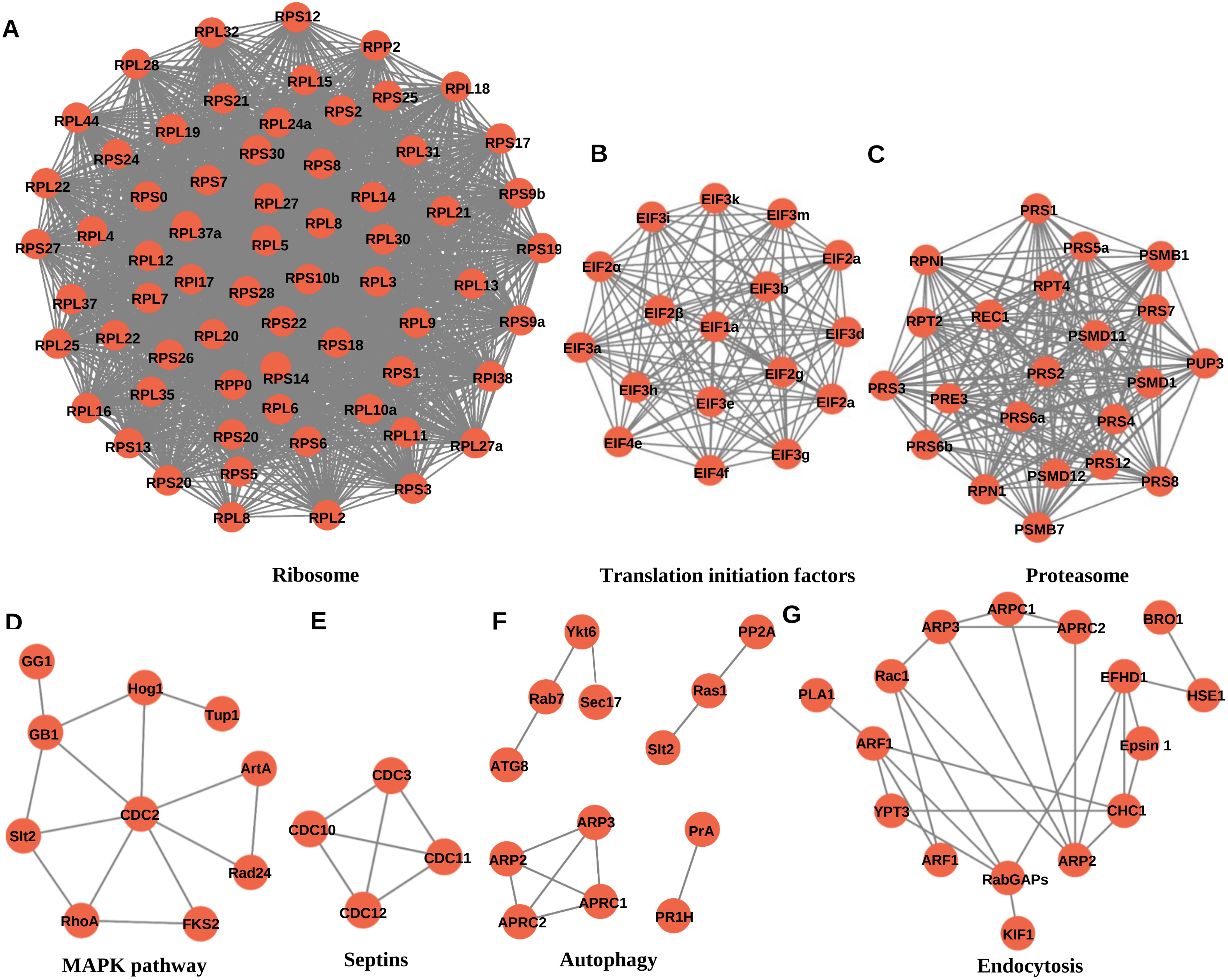
A
RPS12
RPL32
RPP2
RPL28
RPL15
RPL18
RPS21
RPS25
RPS2
RPL44
RPL19
RPL24a
RPS17
RPS24
RPS30
RPS8
RPL31
RPL22
RPS7
RPS0
RPL27
RPL14
RPS9b
RPL21
RPL8
RPL4
RPL37a
RPL30
RPS27
RPL5
RPS19
RPL12
RPS10b
RPI17
RPL3
RPL13
RPL37
RPL7
RPS28
RPS22
RPL9
RPS9a
RPL25
RPL22
RPL20
RPS18
RPS26
RPS14
RPS1
RPI38
RPL35
RPP0
RPL16
RPL6
RPL10a
RPL11
RPS20
RPS6
RPS13
RPL27a
RPS20
RPS5
RPS3
RPL8
RPL2
Ribosome
B
EIF3k
EIF3i
EIF3m
EIF2α
EIF2a
EIF3b
EIF2β
EIF1a
EIF3d
EIF3a
EIF2g
EIF3h
EIF3e
EIF2a
EIF4e
EIF3g
EIF4f
Translation initiation factors
C
PRS1
PRS5a
PSMB1
RPNI
RPT4
RPT2
REC1
PRS7
PSMD11
PUP3
PRS2
PSMD1
PRS3
PRE3
PRS6a
PRS4
PRS6b
PRS12
PSMD12
RPN1
PRS8
PSMB7
Proteasome
D
GG1
Hog1
Tup1
GB1
ArtA
CDC2
Slt2
Rad24
RhoA
FKS2
MAPK pathway
E
CDC3
CDC10
CDC11
CDC12
Septins
F
Ykt6
PP2A
Rab7
Sec17
Ras1
Slt2
ATG8
ARP3
PrA
ARP2
APRC1
APRC2
PR1H
Autophagy
G
ARPC1
ARP3
APRC2
BRO1
Rac1
EFHD1
PLA1
HSE1
ARF1
Epsin 1
YPT3
CHC1
ARF1
ARP2
RabGAPs
KIF1
Endocytosis
