# Supplementary material for: Post-Translational Modification β-Hydroxybutyrylation Regulates Ustilaginoidea virens Virulence
Source: Mol Cell Proteomics. 2023 Jul 12;22(8):100616. doi: 10.1016/j.mcpro.2023.100616 (PMC10423879; doi:10.1016/j.mcpro.2023.100616)
Supplement: Supplemental Figure S8 — Homology analysis of Cdc10p in M. oryzae and U. virens. Amino acid sequence alignment of UvCdc10 and MoSep4. Red highlights indicate the conserved regions of these proteins. [file mmc8.pdf]

```
UvCdc10    1  MAAT.ANTIFPKSHVGFDSITSQIEKKLLKRGFQFNVICVGQTGLGKSTLINTIFASHLI
MoSep4     1  MAAMPAATIYPQSHVGFDSITSQIERKLLKRGFQFNVICVGQTGLGKSTLINTIFASHLI

UvCdc10   60  DSKGRLQPDEAIRSTTEIQSVSHIIEENGVRLRLNIVDTPGYGDLVNNDRCWDPIVKYIK
MoSep4    61  ETKGRHHPDEVIRSTTEIHPVSHIIEENGVRLRLNIVDTPGYGDLINNDRCWDPIVKYIK

UvCdc10  120  DQHSAYLRKELTAQRERYIQDTRIHCCLFFIQPSGHSLKPIDIVVLKKLSDVVNVVPVIA
MoSep4   121  DQHSAYLRKELTAQRERYIQDTRIHCCLFFIQPSGHSLKPIDIVVLKKLSDVVNVVPVIA

UvCdc10  180  KADSLTVEERQAFKERIKEEFAFHNLKMYPYDNEEFDDEERSLNGQIKGLVPFAVVGSEK
MoSep4   181  KADSLTLEERQAFKERIKEEFAFHNLKMYPYDNDDLDEDERSHNQQIKATVPFAVVGSEK

UvCdc10  240  SIMIDGKQVRGRQNRWGVINVEDENHCEFVYLRNFLLRTHLQDLIETTSQIHYETFRAKQ
MoSep4   241  SIIVNGKQVRGRQNRWGVINVEDEEHCEFVHLRNFLLRTHLQDLIETTSQIHYETFRTKQ

UvCdc10  300  LLALKESSAHVGGASSRPISPAADRELSRNSQRMAMNGY
MoSep4   301  LLALKDPSAQ..GHSSRPISPAAEREMSRSSQRMTMNGY
```
